# The Role of Hemosiderin-Laden Macrophages (HLMs) in the Metastasis of Mammary Gland Cancers in Bitches

**DOI:** 10.3390/life16010053

**Published:** 2025-12-29

**Authors:** Kacper Żebrowski, Małgorzata Kandefer-Gola, Izabela Janus-Ziółkowska, Rafał Ciaputa, Stanisław Dzimira

**Affiliations:** Department of Pathology, Division of Pathomorphology and Veterinary Forensics, Faculty of Veterinary Medicine, Wroclaw University of Environmental and Life Sciences, 50-375 Wroclaw, Poland; malgorzata.kandefer-gola@upwr.edu.pl (M.K.-G.); izabela.janus-ziolkowska@upwr.edu.pl (I.J.-Z.); rafal.ciaputa@upwr.edu.pl (R.C.); stanislaw.dzimira@upwr.edu.pl (S.D.)

**Keywords:** hemosiderin, macrophages, dog, mammary gland carcinoma, hemosiderin laden macrophages

## Abstract

Mammary gland tumors are among the most common neoplasms in female dogs. Macrophages are believed to play an important role in tumor progression and metastasis. The aim of this study was to determine whether hemosiderin-laden macrophages (HLMs) may be involved in the development of metastatic lesions in regional lymph nodes. Forty-two cases of mammary gland cancers in female dogs and their regional lymph nodes were included in the analysis. The samples were divided into two groups based on the presence or absence of metastases. The sections were stained with hematoxylin and eosin (HE) and Prussian blue and were additionally subjected to immunohistochemical labeling using antibodies against pan-cytokeratin (Pan-CK) and Ki-67. In 20 cases, no metastatic changes were detected in the regional lymph node, whereas metastases were identified in 22 cases. A positive correlation was observed between the number of HLMs in the tumor stroma and the number of HLMs in the regional lymph node. Furthermore, a positive correlation was found between Ki-67 nuclear immunoreactivity in the mammary tumor and the number of HLMs present within its stroma. HLMs may represent a component of the tumor microenvironment that promotes cancer cell proliferation and potentially contributes to the propensity for metastasis formation in mammary tumors of female dogs.

## Simple Summary

Mammary gland cancer is one of the most common cancers in female dogs, and its spread to regional lymph nodes often worsens the prognosis. Cells of the immune system, called macrophages, are known to influence how cancers grow and spread. Some macrophages contain iron deposits and are known as hemosiderin-laden macrophages. In this study, we examined mammary tumors and regional lymph nodes from dogs with and without cancer spread. We observed that iron-containing macrophages were present both in tumors and in lymph nodes and that their numbers were related to how actively tumor cells were dividing. This study helps to improve understanding of the tumor environment in canine mammary cancer and highlights the possible involvement of iron-containing immune cells in this disease.

## 1. Introduction

Macrophages are immune cells derived from circulating blood monocytes. They reside in nearly all tissues, where they perform a wide range of functions, including phagocytosis of pathogens and apoptotic cells, secretion of cytokines and chemokines, antigen presentation to other immune cells, and participation in tissue repair processes [1].

Macrophages also play a critical role in the tumor microenvironment (TME). Within this context, they are referred to as tumor-associated macrophages (TAMs). TAMs are extensively studied, particularly for their involvement in cancer metastasis [2]. Several mechanisms have been identified through which TAMs promote metastatic progression, including facilitating tumor-cell invasion into surrounding tissues, supporting tumor vascularisation, enabling tumor-cell intravasation via the release of cathepsins, and assisting tumor cells in extravasation from blood vessels [3,4,5,6,7,8,9,10,11].

Depending on the signals present within the tissue microenvironment, macrophages exhibit substantial polarization plasticity. Based on their polarization state, they are commonly classified as M1 or M2 macrophages [12]. M0 macrophages, which represent the undifferentiated precursors of both M1 and M2 subsets, can also be distinguished [1]. M1 macrophages are generated through classical activation. They play a pivotal role in combating infections and orchestrating pro-inflammatory immune responses [13]. Polarization towards the M1 phenotype is induced by pro-inflammatory cytokines—such as interferon-γ (IFN-γ) and tumor necrosis factor-α (TNF-α)—as well as immunostimulatory cytokines including IL-12 [14]. Alternatively, macrophages may undergo polarization towards the M2 phenotype. M2 macrophages are polarized under the influence of anti-inflammatory cytokines (IL-4, IL-10 and IL-13). These macrophages contribute to the suppression of inflammatory responses and promote tumor progression by attenuating the host immune response, enhancing angiogenesis, and increasing tumor-cell motility [14,15,16]. It is important to note that macrophage polarization is not a fixed state but a dynamic and reversible process that reflects ongoing changes within the tumor microenvironment [17].

One of the key functions of macrophages in the body is their active involvement in iron metabolism. They recover iron from aging or damaged red blood cells as well as from destroyed tissues [18,19,20]. Macrophages that contain hemosiderin within their cytoplasm, in the form of aggregated ferritin granules, are referred to as hemosiderin-laden macrophages (HLMs). Importantly, HLMs may exhibit either M1 or M2 polarization, and these subsets differ in their mechanisms of iron handling [21].

Mammary gland tumors are the most frequently diagnosed neoplasms in female dogs [22,23]. These tumors display substantial morphological variability and biological heterogeneity within the host organism [22]. The majority are malignant and exhibit a marked tendency to metastasize. The most common metastatic sites include regional lymph nodes and the lungs, whereas metastasis to other organs is comparatively rare [24]. In most cases, metastatic lesions and their detrimental impact on the function of affected organs are the primary cause of death [25].

This study aims to investigate whether a relationship exists between the presence, number, and distribution of HLMs in mammary tumors in female dogs and the occurrence of metastases in the regional lymph nodes. Additionally, the study will assess the presence, number, and spatial distribution of HLMs within these lymph nodes.

## 2. Materials and Methods

The study included 42 cases of mammary gland cancers in female dogs, along with their regional lymph nodes. Each case concerns a dog diagnosed with a confirmed primary mammary gland carcinoma. The sample size for the finite population (canine mammary gland cancers) was calculated using the biostat calculator, considering the assumed level of statistical significance (0.05), the size of the dog population in Wrocław, and the acceptable error. The material was obtained from the archives of the Department of Pathology at the University of Environmental and Life Sciences in Wrocław. Tumors collected between 2021 and 2024 were selected for analysis. Selection criteria were based on breed and age data available in the archival database. The average age of the animals was ten and one-half years (range: 3–16 years). Table 1 summarizes the breeds of the dogs included in the study and the number of individuals per breed.

### 2.1. Hematoxylin and Eosin (HE)

All samples were fixed in 10% neutral buffered formalin for 24 h, followed by routine histological processing and embedding in paraffin wax. From each paraffin block, serial sections 3 µm in thickness were prepared. One section was stained with HE, while the remaining sections were allocated for histochemical and immunohistochemical analyses.

Based on HE-stained sections, tumors were classified according to the system developed by Goldschmidt et al. [26], and the presence or absence of metastatic lesions in the corresponding lymph nodes was confirmed.

In addition, HE staining was used to assess tumor malignancy grade. For this purpose, the grading system proposed by Elston and Ellis [27] and subsequently modified by Peña et al. [28] was applied.

### 2.2. Prussian Blue

Perls staining was performed using a commercial kit from Elektromed (Zabierzów Bocheński, Poland, DP-010236). Briefly, formalin-fixed, paraffin-embedded sections were deparaffinised and rehydrated, after which they were incubated with Perls reagent (a 1:4 mixture of 50% HCl and 2% potassium ferrocyanide) for 30 min at room temperature. The slides were then rinsed in distilled water. Nuclear contrast staining was carried out using Kernechtrot (Nuclear Fast Red), followed by a second rinse in distilled water for two minutes. A spleen section served as the positive control.

Within the tumor tissue, the number of blue-stained macrophages was counted in five hot-spot areas—places with the highest number of HLMs, including both the tumor stroma and the peritumoral region. The area of a single hot-spot was 0.16 mm^2^, making the total area of the five hot-spots 0.8 mm^2^. In the lymph node sections, the five areas with the highest number of HLMs were evaluated. The total number of positive cells per case was counted as the sum of five hot-spots. Their localization was also recorded and classified into one of the following regions: cortex, medulla, or both cortex and medulla of the lymph node.

The density of HLMs was calculated by dividing the number of HLMs in the five hot-spots by the total area (in mm^2^), and the results were expressed as cells/mm^2^.

### 2.3. Immunohistochemistry

Immunohistochemical analyses were performed on canine mammary tumors and lymph nodes using a Leica Bond-Max automated stainer (Leica Biosystems, Newcastle, UK) according to the following protocol. Tissue sections were first deparaffinised with Bond Dewax Solution (Leica Biosystems, UK) and subjected to heat-induced epitope retrieval using Bond Epitope Retrieval Solution 1 (Leica Biosystems, UK) for 20 min. Endogenous peroxidase activity was blocked using the BOND Polymer Refine Detection System (Leica Biosystems, UK) with Peroxide Block. Primary antibodies used in this study included Ki-67 (clone MIB-1, M0821, Dako, Glostrup, Denmark), diluted 1:100 in Bond Primary Antibody Diluent (Leica Biosystems, UK), and Pan-CK (clone MNF 116, M7240, Dako, Denmark), diluted 1:75 in the same diluent. Sections were incubated with primary antibodies for 15 min at room temperature. Subsequently, the samples were incubated with the post-primary reagent and polymer using the BOND Polymer Refine Detection System (Leica Biosystems, UK). 3,3′-Diaminobenzidine (DAB) served as the chromogen, after which the slides were counterstained with hematoxylin (BOND Polymer Refine Detection System, Leica Biosystems, UK).

Negative controls were prepared by replacing the primary antibody with Bond Primary Antibody Diluent (Leica Biosystems, UK). Rabbit serum, matched in protein concentration to the primary antibody, was used in place of the primary antibody as a negative control to assess staining specificity. For Ki-67, lymph node tissue served as the positive control, while skin fragments were used as the negative control. For Pan-CK, healthy mammary gland tissue was used as the positive control. Spleen tissue was used as the negative control, as epithelial tissue is not normally present in this organ.

The Ki-67 reaction was used to assess the proliferative activity of tumor cells, and in this context, it was applied to evaluate whether HLMs contribute to malignant tumor development by increasing cancer-cell proliferation. Quantification of the Ki-67 reaction was performed by counting positively stained nuclei in five hotspot areas within the tumor and, when applicable, within metastatic lesions in the lymph node. The total number of positive cells per case was counted as the sum of five hot-spots. Additionally, the number of cell nuclei within the primary tumor and the metastatic lesion in the regional lymph node was counted in five hot-spots, after which the percentage of nuclei showing a positive reaction was calculated. The density of the Ki-67 reaction was calculated by dividing the number of positively stained nuclei in the five hotspots by the total area (in mm^2^), and the results were expressed as nuclei/mm^2^.

The Pan-CK reaction in lymph node sections was used to confirm or exclude the presence of metastatic mammary carcinoma in the examined animals.

### 2.4. Microscopic Photographs

The number of cells that were positive for Prussian blue staining and immunohistochemistry (for Ki-67) was determined by examining photographs of preparations taken at 400× magnification. Computer-assisted image analysis was performed on microscopic images of mammary gland tumor sections using a computer connected to an Olympus BX53 optical microscope (Olympus, Tokyo, Japan) equipped with a digital Olympus UC90 camera and CellSens Standard v.1 software (Olympus, Tokyo, Japan).

### 2.5. Statistical Analysis

Statistical analysis was performed using Statistica 13.3 (TIBCO Software Inc., Palo Alto, CA, USA) and appropriately selected statistical tests. Normality of data was tested using the Shapiro–Wilk test. The comparison of HLMs number in various tumor types, tumor malignancy grade and of the metastasis presence in lymph nodes with various HLMs localization was tested using Kruskal–Wallis analysis with Dunn post hoc test. The comparison of HLMs number in tissues with or without metastases was tested using Mann–Whitney U analysis. The correlation between the HLMs number in tumor tissue and lymph nodes, between the HLMs number and Ki-67 expression, and between Ki-67 expression in tumor and lymph node was tested using Spearman’s correlation analysis with the correlation strength assessed as poor, fair, moderate, strong or perfect as proposed by Akoglu [29]. The significance level was set at *p* ≤ 0.05.

## 3. Results

### 3.1. HE

All cases selected for analysis were diagnosed as carcinomas of mammary gland origin based on HE staining. The following histological types were identified: 24 simple tubular carcinomas, 5 mixed-type carcinomas, 3 complex-type carcinomas, 3 solid carcinomas, 3 simple tubular-papillary carcinomas, 2 simple cystic-papillary and 2 intraductal papillary carcinomas.

Of the 42 tumors, 13 were classified as grade I, 16 as grade II, and 13 as grade III in terms of histological malignancy. The distribution of carcinoma types according to their malignancy grade is presented in Table 2.

Metastatic lesions in the regional lymph nodes were absent in 20 of the 42 tumors, whereas the remaining 22 cases exhibited metastatic involvement (Table 2).

### 3.2. Prussian Blue

**Figure 1:**
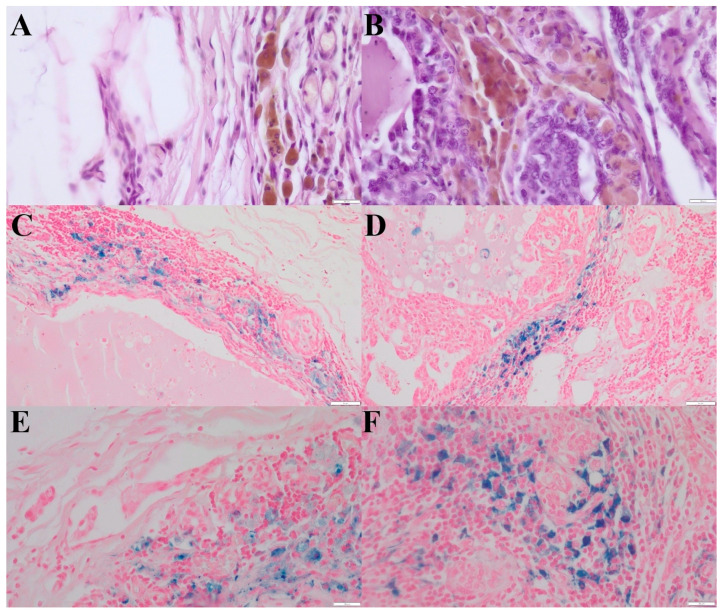
(**A**)—HLMs in the peritumoral region of simple tubular carcinoma, grade III malignancy. Aggregates of hemosiderin have brown color. (HE, 400×). (**B**)—HLMs within the tumor stroma of simple tubular carcinoma, grade III malignancy. Aggregates of hemosiderin have brown color. (HE, 400×). (**C**)—HLMs in the peritumoral region of simple tubular carcinoma, grade III malignancy (Prussian blue, 200×). (**D**)—HLMs within the tumor stroma of simple tubular carcinoma, grade III malignancy (Prussian blue, 200×). (**E**)—Higher magnification of HLMs in the peritumoral region of simple tubular carcinoma, grade III malignancy. (Prussian blue, 400×). (**F**)—Higher magnification of HLMs within the tumor stroma of simple tubular carcinoma, grade III malignancy (Prussian blue, 400×). The scale bars in the above images represent: (**A**,**B**,**E**,**F**): 20 μm; (**C**,**D**): 50 μm.

In cases of tumors without lymph node metastases, the average number of HLMs counted in five fields of view was 59 (range: 0–196) in the tumor stroma and 21 (range: 0–93) in the peritumoral region (Figure 1). In the regional lymph nodes, the average number of HLMs in five fields of view was 108 (range: 10–200).

The average density of HLMs counted in five hot-spots of these tumors was 74 cells/mm^2^ in the tumor stroma and 26 cells/mm^2^ in the peritumoral region. The average density of HLMs in the regional lymph node was 135 cells/mm^2^.

Among the 20 lymph nodes without metastatic lesions, HLMs were located solely in the cortex in four cases, solely in the medulla in 12 cases, and evenly distributed between the cortex and medulla in four cases.

**Figure 2:**
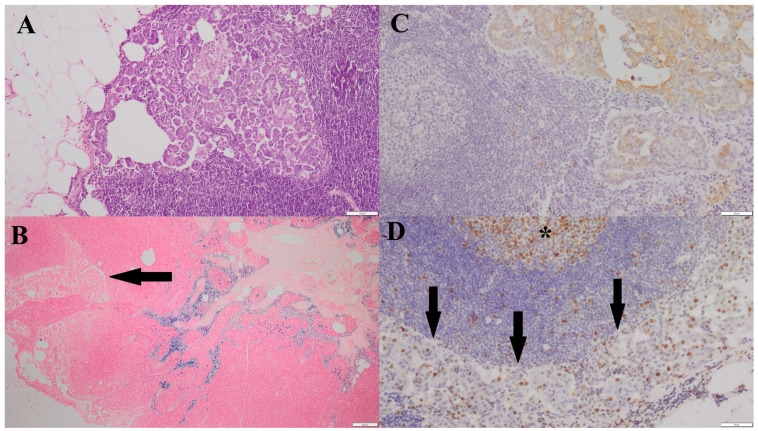
(**A**)—Metastatic focus of simple tubular carcinoma, grade III malignancy, located in the cortex of the associated lymph node (HE, 100×). (**B**)—HLMs within the medulla of the lymph node containing a metastatic focus; a metastatic lesion is visible in the cortical region (arrow) (Prussian blue, 40×). (**C**)—Positive Pan-CK immunoreactivity within the metastatic focus in the cortex of the associated lymph node (200×). (**D**)—Ki-67 nuclear immunoreactivity in the cells of the metastatic lesion (arrow); Ki-67–positive nuclei are visible within the center of a lymphoid follicle of the lymph node (*) (200×).

In tumors with metastatic involvement of the lymph node, the average number of HLMs counted in five fields of view was 66 (range: 0–166) in the tumor stroma and 23 (range: 0–100) in the peritumoral region (Figure 2A,B). In the corresponding lymph nodes, the average number of HLMs in five fields of view was 150 (range: 50–350).

The average density of HLMs counted in five hot-spots of tumors with metastatic changes was 83 cells/mm^2^ in the tumor stroma and 29 cells/mm^2^ in the peritumoral region. The average density of HLMs in the regional lymph node was 188 cells/mm^2^.

In 4 of the 22 lymph nodes containing metastases, HLMs were located in the cortex, in 14 cases they were in the medulla, and in 4 cases they were distributed evenly between the cortex and medulla.

A positive correlation was observed between the number of HLMs in the tumor stroma and the number of HLMs in the corresponding lymph node (Spearman’s test; r = 0.37, *p* < 0.05).

No significant differences were found between the number of HLMs in the tumor stroma, peritumoral region, or associated lymph nodes and the histological type of mammary carcinoma (Kruskal–Wallis test, *p* > 0.05), the degree of histological malignancy (Kruskal–Wallis test, *p* > 0.05), or the presence of metastases in the associated lymph node (Mann–Whitney U test, *p* > 0.05) (Table 3).

Additionally, no difference was observed between the presence of metastatic lesions in the lymph node and the localization of HLMs within the lymph node (cortex, medulla, or both) (Kruskal–Wallis test, *p* > 0.05).

### 3.3. Immunohistochemistry

A positive cytoplasmic reaction for Pan-CK was observed in 22 of the 42 lymph node sections (Figure 2C), confirming the presence of metastatic lesions of epithelial origin in all cases previously identified by HE staining. In the remaining 20 cases, the absence of cytokeratin staining confirmed the lack of metastatic lesions in the regional lymph nodes.

The Ki-67 nuclear labeling index, evaluated in five hotspots at 400× magnification, averaged 40 (range: 4–150) in carcinomas without lymph node metastases. In carcinomas with confirmed lymph node metastases, the average Ki-67 index was 62 (range: 7–150). Within metastatic foci in the lymph node, the average Ki-67 index was 23 (range: 5–160) (Figure 2D; Table 4).

The average number of cell nuclei within the primary tumor and the metastatic lesion in the regional lymph node was 440 in five hot-spots. Consequently, the percentage of Ki-67–positive nuclei was 9.09% (40/440) in carcinomas without lymph node metastasis, 14.09% (62/440) in carcinomas with confirmed metastasis, and 5.23% (23/440) in metastatic foci in the regional lymph node.

The average density of the Ki-67 reaction counted in five hot-spots of tumors without metastatic changes was 50 nuclei/mm^2^, while in tumors with metastatic changes it was 76 nuclei/mm^2^. In the metastatic foci within the regional lymph node, the average Ki-67 density was 29 nuclei/mm^2^.

A positive correlation was observed between the Ki-67 nuclear labeling index in mammary tumors and the number of HLMs in the tumor stroma (Spearman’s test; r = 0.37, *p* < 0.05). However, no significant correlation was found between Ki-67 in the tumor and the number of HLMs in the peritumoral region or in the regional lymph node.

Additionally, a positive correlation was detected between the Ki-67 index in the primary tumor and the Ki-67 index in metastatic lesions within the associated lymph node (Spearman’s test; r = 0.43, *p* < 0.05).

## 4. Discussion

Macrophages, including specialized populations known as TAMs, play a crucial role in the tumor microenvironment and can influence metastasis at multiple levels [30]. Their involvement includes enhancing cancer cell motility [10,11], supporting epithelial–mesenchymal transition (EMT) [4,9,31,32,33], preparing the pre-metastatic niche (PMN) in distant organs [5,8], and promoting angiogenesis [3,6]. Collectively, these processes form a complex network of interactions that may facilitate the colonization of distant tissues by cancer cells.

The literature also highlights the critical role of macrophages in iron metabolism, which directly affects cellular function within the tumor microenvironment (TME). M1-polarized macrophages tend to sequester iron, thereby limiting its availability to cancer cells [17]. In contrast, M2-polarized macrophages, which are generally associated with reparative and immunosuppressive functions, exhibit increased expression of ferroportin [34], promoting the release of iron into the extracellular space. Elevated iron availability can exacerbate oxidative stress and activate proliferative signaling pathways, including the mitogen-activated protein kinase (MAPK) cascade [35], thereby promoting tumor growth and increasing the risk of metastasis formation.

HLMs represent a distinct subpopulation of macrophages, combining the functions described above with a specialized role in iron recycling. To date, HLMs have been primarily studied in the context of respiratory and cardiovascular diseases [36,37,38,39,40,41,42,43], whereas their role in oncology, both in humans and animals, remains poorly understood.

The observed correlation between the number of HLMs in the tumor stroma and proliferative activity, as assessed by the Ki-67 marker, suggests that HLMs may support cancer cell proliferation. This effect may result, in part, from the provision of iron to tumor cells or from the immunosuppressive properties associated with an M2-like macrophage phenotype. The findings of Giambrone et al., who reported the expression of M2-associated markers—including CD204, vascular endothelial growth factor (VEGF), vascular endothelial growth factor receptor-1 (VEGFR-1), and transforming growth factor alpha (TGF-α)—in HLMs [44], further support the hypothesis that these cells exhibit protumorigenic activity. Furthermore, the literature indicates that a higher number of TAMs is associated with the invasion and progression of canine mammary tumors, and that there is a shift from M1 macrophages in benign tumors to M2 macrophages in malignant tumors [45]. Furthermore, a positive correlation has been demonstrated in human medicine between the presence of numerous macrophages and increased Ki-67 expression in female breast cancers [46].

An important aspect of this study was the analysis of the relationship between the presence of HLMs in the primary tumor and their occurrence in the regional lymph node. The results demonstrated a positive correlation between these parameters, which may support the hypothesis that HLMs contribute to the formation of a pre-metastatic microenvironment. Previous studies indicate that macrophages can migrate via the blood or lymphatic system and modulate the lymph node environment through the release of cytokines, growth factors, and iron metabolites [5,8,35]. The role of macrophages in preparing the PMN had also been described in human medicine, where the studies prove that M1 and M2 macrophages have different impact on this process [47]. Our findings suggest that a similar process may occur in mammary gland tumors in female dogs, but further extensive research is required in this area.

No statistically significant correlation was observed in this study between the histological type of the tumor and the number of HLMs in the analyzed locations, including the tumor stroma, peritumoral region, and lymph nodes. Giambrone et al. reported differences in the distribution and number of HLMs in mammary tumors of female dogs [44]. In various subtypes of mammary gland cancer in female dogs, the number of HLMs showed statistically significant differences in terms of their total number within tumors and their number exclusively in the tumor stroma. HLMs occurred less frequently in the stroma of complex carcinomas. The authors acknowledged that the small sample size limited their results, meaning that their correlations are speculative. In the available literature, the involvement of HLMs in human breast cancer has been rarely investigated [48,49,50]. Lee et al. and Pastorello et al. reported a higher incidence of HLMs in the stroma of triple-negative breast cancer (TNBC) [48,49]. Their findings did not establish a definitive role for HLMs in the metastatic process, the presence of these cells in more aggressive tumor subtypes suggests a potential contribution to tumor progression.

Additionally, in this research, no correlation was also observed between histological malignancy and the number of HLMs. A lack of correlation with histological malignancy has also been reported in the aforementioned study [44]. Therefore, this lack of correlation could be attributed to the heterogeneity of mammary tumors in dogs, as well as the limited sample size. It can also be assumed that in more malignant tumors, iron is localized subcellularly. This is likely due to cell breakdown in necrotic areas, which are characteristic of tumors with a higher degree of malignancy. This may explain why we did not observe an increased number of HLMs in highly malignant tumors [51]. All these factors should be taken into account when interpreting the results.

This study represents one of the first comprehensive assessments of the correlation between the presence of HLMs in mammary tumors and regional lymph nodes in female dogs. Although some of the observed results did not reach statistical significance, the trends identified justify further in-depth investigations, including broader panels of immunohistochemical markers, functional characterization of HLMs, and evaluation of their role in modulating iron metabolism within the tumor microenvironment.

This research had certain limitations. First, the study included a relatively small number of tumors, as not all of the samples delivered to our facility were accompanied by the regional lymph node. Another limitation was that the study involved different types of mammary gland cancer in female dogs. Due to the high morphological diversity of mammary gland tumors in female dogs, it was difficult to obtain a sufficient number of cases of a single tumor type together with the corresponding lymph node.

## 5. Conclusions

HLMs are associated with proliferative activity (Ki-67) and show a modest correlation between primary tumor stroma and regional lymph nodes, suggesting a potential role in microenvironmental modulation. However, HLM counts did not differ significantly between metastasis-positive and metastasis-negative cases. Also non-statistical significance was found in the number of HLMs in the peritumoral region vs. in regional lymph nodes. The specific role of HLMs in metastasis requires further investigation.

In future studies, it would be valuable to expand the immunohistochemical panel to include markers characteristic of M1 and M2 macrophages (CD68, CD163, CD204, CD206) as well as markers of angiogenesis and immunosuppression (VEGF, VEGFR-1, TGF-α). This approach would enable a more precise characterization of HLM polarization and function within the tumor microenvironment. Additionally, increasing the sample size and evaluating the interactions between HLMs and other components of the microenvironment, such as fibroblasts, lymphocytes, and the vascular and lymphatic networks, will be important for a more comprehensive understanding of their role in tumor progression.

## Figures and Tables

**Table 1:** Dog breeds included in the study.

Dog Breed	Number
Mixed breed	23
Yorkshire terrier	6
Shih tzu	3
Maltese	2
German shepherd	2
American Staffordshire terrier	1
Polish greyhound	1
Chihuahua	1
Dachshund	1
Kerry blue terrier	1
Bavarian mountain hound	1

**Table 2:** Classification of mammary gland carcinomas included in the study by histological subtype and malignancy grade.

Type of Cancer	No Metastases in the Lymph Nodes		Presence of Metastases in Lymph Nodes	
	Degree of Malignancy	Number	Degree of Malignancy	Number
Simple tubular carcinoma	I	9	I	0
	II	3	II	5
	III	0	III	7
Mixed-type carcinomas	I	1	I	0
	II	3	II	0
	III	0	III	1
Complex-type carcinoma	I	0	I	0
	II	1	II	0
	III	1	III	1
Solid carcinoma	I	1	I	0
	II	0	II	0
	III	0	III	2
Simple tubular-papillary carcinoma	I	1	I	0
	II	0	II	2
	III	0	III	0
Simple cystic-papillary carcinoma			I	0
			II	2
			III	0
Intraductal papillary carcinoma			I	0
			II	1
			III	1
Summary		20		22

**Table 3:** Number of HLMs and differences between evaluated parameters.

	Tumor Stroma	Peritumoral Region	Regional Lymph Node
Type of cancer			
Simple tubular carcinoma	52 (0–196)	18 (0–93)	106 (10–160)
Mixed-type carcinomas	65 (18–95)	23 (0–52)	150 (80–250)
Complex-type carcinoma	102 (70–166)	48 (0–81)	96 (37–130)
Solid carcinoma	92 (70–125)	43 (0–30)	127 (80–200)
Simple tubular-papillary carcinoma	98 (50–125)	25 (0–45)	217 (150–250)
Simple cystic-papillary carcinoma	73.5 (62–85)	4 (0–8)	225 (200–250)
Intraductal papillary carcinoma	33 (25–41)	11 (0–21)	225 (100–350)
Degree of malignancy			
I	60 (0–196)	15 (0–93)	100 (10–160)
II	55 (0–125)	28 (0–81)	149 (50–250)
III	75 (0–166)	21 (0–100)	138 (80–350)
Presence of metastases in regional lymph node			
No	60 (0–196)	21 (0–93)	108 (10–200)
Yes	66 (0–166)	23 (0–100)	150 (60–350)

Table showing the average number of HLMs in five hot-spot areas, including minimum and maximum values.

**Table 4:** Ki-67 index values in mammary gland cancers in bitches with and without metastasis, according to the degree of malignancy.

Ki-67 Index	
Tumor without metastasis	40 (4–150)
Tumor with metastasis	62 (7–150)
Metastatic foci in the lymph node	23 (5–160)
Ki-67 index in terms of degree of malignancy	
I	30 (4–115)
II	67 (4–150)
III	55 (16–150)

Table showing the average index of Ki-67 in five hot-spots areas, with minimum and maximum values.

## Data Availability

The datasets used and/or analyzed during the current study are available from the corresponding author upon reasonable request.

## Abbreviations

The following abbreviations are used in this manuscript:

HLMs	hemosiderin-laden macrophages
HE	hematoxylin and eosin
TME	tumor microenvironment
TAMs	tumor-associated macrophages
IFN-γ	interferon gamma
TNF-α	tumor necrosis factor alpha
Pan-CK	pan-cytokeratin
PMN	pre-metastatic niche
EMT	epithelial–mesenchymal transition
MAPK	mitogen-activated protein kinases
TNBC	triple-negative breast cancer
VEGF	vascular endothelial growth factor
VEGFR-1	vascular endothelial growth factor receptor-1
TGF-α	transforming growth factor alpha
